# Adolescent but not adult-born neurons are critical for susceptibility to chronic social defeat

**DOI:** 10.3389/fnbeh.2014.00289

**Published:** 2014-08-28

**Authors:** Greer S. Kirshenbaum, Sophie R. Lieberman, Tamara J. Briner, E. David Leonardo, Alex Dranovsky

**Affiliations:** Dranovsky-Leonardo Lab, Department of Psychiatry, Division of Integrative Neuroscience, New York State Psychiatric Institute, Columbia UniversityNew York, NY, USA

**Keywords:** adolescent, neurogenesis, GFAP-Tk, chronic social defeat, resilient

## Abstract

Recent evidence implicates adult hippocampal neurogenesis in regulating behavioral and physiologic responses to stress. Hippocampal neurogenesis occurs across the lifespan, however the rate of cell birth is up to 300% higher in adolescent mice compared to adults. Adolescence is a sensitive period in development where emotional circuitry and stress reactivity undergo plasticity establishing life-long set points. Therefore neurogenesis occurring during adolescence may be particularly important for emotional behavior. However, little is known about the function of hippocampal neurons born during adolescence. In order to assess the contribution of neurons born in adolescence to the adult stress response and depression-related behavior, we transiently reduced cell proliferation either during adolescence, or during adulthood in GFAP-Tk mice. We found that the intervention in adolescence did not change adult baseline behavioral response in the forced swim test, sucrose preference test or social affiliation test, and did not change adult corticosterone responses to an acute stressor. However following chronic social defeat, adult mice with reduced adolescent neurogenesis showed a resilient phenotype. A similar transient reduction in adult neurogenesis did not affect depression-like behaviors or stress induced corticosterone. Our study demonstrates that hippocampal neurons born during adolescence, but not in adulthood are important to confer susceptibility to chronic social defeat.

## Introduction

Neurogenesis, the birth and integration of new neurons, occurs in the hippocampus across the lifespan. Hippocampal neurogenesis in humans has been observed to progress at a relatively constant rate and may modestly decline after adolescence (Knoth et al., 2010; Spalding et al., 2013). In mice, neurogenesis is most prevalent in the hippocampus at birth and significantly declines across postnatal development. In fact, the majority of the hippocampal dentate gyrus (DG) is not formed until late gestation and early post-natal life (Seki et al., 2014). By early adolesence, or postnatal day 28 (P28) in mice, neurogenesis is restricted to the subgranular zone (SGZ) of the DG. During this time, newborn neurons populate the same anatomical space as cells born during adulthood, but at a considerably (up to 300%) higher rate (He and Crews, 2007). Finally in adulthood, which begins at approximately postnatal day 56 (P56), neurogenesis continues in the SGZ, but at a lower and continuously declining rate throughout life (Zhao et al., 2008; Li et al., 2009). In adolescent and adult mice SGZ neural precursors differentiate into new neurons and functionally integrate into the granule cell layer (Toni et al., 2008). Although the process and localization of neurogenesis is homologous in adolescent and adult mice, the connectivity and functional contribution of new neurons may differ at these different stages of life (Wei et al., 2011).

Adolescence is a sensitive period in development wherein stressful experience can either increase risk for depressive disorders or promote resiliency (Buwalda et al., 2011). Evidence suggests that experience during development changes the brain in order to prepare an organism to respond optimally during adulthood (Nederhof and Schmidt, 2012). Brain circuitry underlying emotional behavior, including in the prefrontal cortex, hippocampus and amygdala, undergoes plasticity and functions differently in adolescence compared to adulthood and may be vulnerable to environmental influence (Pattwell et al., 2011; Selemon, 2013). For instance the expression of fear in a contextual fear-conditioning paradigm is strikingly suppressed during adolescence from P29-P33 in mice, reflecting a transient alteration of hippocampal and amygdalar function (Pattwell et al., 2011). Moreover adolescents may experience stressful stimuli differently than adults; activation of the hypothalamic pituitary adrenal axis during adolescence takes twice as long to recover to baseline following stress in rats (Romeo, 2010). Remarkably, SGZ neurogenesis plays a role in the stress response (Snyder et al., 2011) and is greatly reduced by stressful experiences (Dranovsky and Hen, 2006). Therefore, suppression of neurogenesis during adolescence, when it is more robust than in adulthood, may have greater consequence for hippocampal function in encoding stress. We hypothesized that neurons born from the large population of adolescent SGZ progenitor cells contribute to the development of emotional brain circuitry during this sensitive period and accordingly granule neurons generated during adolescence differentially contribute to mature circuits related to depression and stress regulation in adulthood.

Studies have shown that adult-born SGZ neurons contribute to regulation of the hypothalamic pituitary adrenal (HPA) axis (Schloesser et al., 2009; Snyder et al., 2011) and stress induced emotional behavior such as susceptibility to and recovery from chronic social defeat (Lagace et al., 2010; Schloesser et al., 2010; Lehmann et al., 2013). Behavioral responses to antidepressant treatment were also linked to adult-born neurons (Santarelli et al., 2003; David et al., 2009). However, the contribution of adult-born neurons to baseline emotional behavior remains unclear (Revest et al., 2009; Wei et al., 2011). Even less is known about the involvement of adolescent-born granule neurons to emotional behavior and stress reactivity. Ablation of neurogenesis throughout adolescence and adulthood in female mice impaired adult social behavior, whereas ablation throughout adulthood did not (Wei et al., 2011). This suggests that neurons arising from adolescent neurogenesis may contribute to specific brain circuitry dictating sociability, and raises the possibility that cells born during adolescence contribute to other emotional circuits. To investigate the contribution of exclusively adolescent-born neurons in the SGZ to depression-related circuitry, we have employed the GFAP-Tk mouse (Bush et al., 1998) and created a valgancyclovir (VGCV) treatment regimen that transiently reduced neurogenesis from P28-P42. We then measured depression and stress-related behavior and physiology in adulthood. To compare the function of adolescent-born and adult-born neurons we also reduced neurogenesis in adult GFAP-Tk mice with VGCV and compared outcomes at a time when all depleted cells would have been mature.

## Materials and methods

### Subjects

GFAP-Tk heterozygous mice (GFAP-Tk^+/−^; Bush et al., 1998) allow us to transiently reduce neurogenesis. These mice express herpes thymidine kinase (Tk) under control of the stem and non-stem astrocyte-specific glial fibrillary acidic protein (GFAP) promoter. Neural progenitor cells can be eliminated in GFAP-Tk^+/−^ mice by treatment with VGCV (Roche), which is phosphorylated by Tk leading to abortive replication in mitotically active cells. Since non-stem astrocytes are seldom dividing, they are spared during gancyclovir treatment (Garcia et al., 2004). Nestin-CreERT2 heterozygous R26R-Stop-EYFP heterozygous mice (Dranovsky et al., 2011) allow us to visualize cumulative levels of neurogenesis across time. Nestin-CreERT2 mice express a Tamoxifen-inducible Cre in stem cells. When tamoxifen is administered it binds to the estrogen receptor, which then translocates Cre to the nucleus allowing it to excise the stop sequence resulting in expression of EYFP in nestin stem cells. Thus nestin stem cells and all of their resulting neuronal and astrocytic progeny express the EYFP protein indelibly.

Female GFAP-Tk heterozygous Nestin-CreERT2 heterozygous R26R-Stop-EYFP homozygous mice were mated with R26R-Stop-EYFP homozygous males, all on C57BL/6J 129S6 mixed background. Male pups were genotyped using previously reported PCR reactions (Bush et al., 1998; Dranovsky et al., 2011), weaned at P21, and housed 3–5 per cage with mixed genotypes. Half the mice were GFAP-Tk heterozygous (GFAP-Tk^+/−^ ) and half were negative for the gene (GFAP-Tk^−/−^), in addition half of each group were Nestin-CreERT2 heterozygous and all were R26R-Stop-EYFP homozygous. Mice were given *ad libitum* access to food and water under a 12:12 h light:dark cycle in a temperature-controlled (72°F) colony. All animal experiments were performed in accordance with the Guide for the Care and Use of Laboratory Animals and approved by the New York State Psychiatric Institute Animal Care and Use Committee.

### Reduction of neurogenesis by valgancyclovir in GFAP-Tk^+/−^ mice

ChowG containing VGCV (165 mg/kg) was administered to mice at P28-P35 in the adolescent group (GFAP-Tk^−/−^ *n* = 25, GFAP-Tk^+/−^ *n* = 27) and at P56-P63 (GFAP-Tk^−/−^ *n* = 21, GFAP-Tk^+/−^ *n* = 29) in the adult group.

### Evaluation of valgancyclovir treatment to transiently reduce neurogenesis in GFAP-Tk^+/−^ mice

Thymidine analogs bromodeoxyuridine (BrdU), 5-chloro-2′-deoxyuridine (CldU) and iododeoxyuridine (IdU) incorporate into the DNA of dividing cells and serve as markers of DNA replication. BrdU was used to label DNA replication at a single time point. Discrete antibodies bind to CldU and IdU (Vega and Peterson, 2005) so the two markers were used to visualize DNA replication at two different historical time points in the same animal.

To assess if VGCV reduced cell proliferation after 7 days of treatment from P28-P35, a cohort of mice was injected with BrdU (50 mg/kg, dissolved in 0.1 M PBS, Roche) intraperitoneally on P35 and P36 and mice were sacrificed on P36. To assess if cell proliferation was restored 7 days following cessation of VGCV treatment, a separate cohort of mice treated with VGCV from P28-P35 was injected with the same dose of BrdU on P42 and P43 and sacrificed on P43.

To assess VGCV mediated reduction in neurogenesis in adolescent and adult mice used in behavioral experiments, CldU (42.5 mg/kg dissolved in 0.1 M PBS, Sigma) was injected after 7 days of VGCV treatment intraperitoneally, on P35 and P36 in the adolescent group and on P63 and P64 in the adult group. To assess if cell proliferation was restored 7 days following cessation of VGCV treatment, mice were injected with IdU (57.5 mg/kg dissolved in 0.1 M PBS with 2% 0.2 M NaOH; MP Biochemicals) intraperitonially on P42 and P43 in the adolescent group and on P70 and P71 in the adult group. Finally to assess if cumulative levels of neurogenesis were normalized following VGCV treatment, mice were treated with 2.5 mg tamoxifen (Sigma) suspended in 1:1 honey water via gavage on P42 for the adolescent group and P70 for the adult group. All mice were sacrificed after behavioral experiments on P130.

### Behavioral experiments

All behavioral experiments were performed by female scientists and took place during the light cycle between 9 AM and 2 PM. Behavioral studies were conducted in adulthood beginning at P90 in the following order: sucrose preference test, social affiliation, forced swim test, stress induced corticosterone, and social defeat. All experiments were performed and scored with experimenters blind to genotype.

### Sucrose preference

An 8-day sucrose preference protocol was performed as previously described (Roybal et al., 2007) with modifications. Mice were group housed and deprived of water in the homecage for 8 days starting 12 h before the beginning of the experiment. Once every 24 h mice were presented with two identical bottles with ball bearing sipper tubes. To avoid a side bias bottle position was alternated daily. The weight of bottles was recorded daily to assess the amount of solution consumed. On days 1 and 2, mice were presented with two identical bottles filled with water (water/water) for 2 h and 1 h respectively. On days 3 and 4, both bottles contained 4% sucrose solution dissolved in the drinking water (sucrose/sucrose) for 1 h and 30 min respectively. On days 5–8, one bottle was filled with water and the other was filled with 4% sucrose solution for 30 min each day (water/sucrose). Preference on each day was calculated as: (weight bottle 1/ (weight bottle 1 + weight bottle 2) ×100). Preference was averaged for each condition (water/water, sucrose/sucrose or water/sucrose).

### Social affiliation

Social affiliation was performed as described with modifications (Nadler et al., 2004). Mice were placed in an empty three chambered apparatus for a 5 min habituation period under 250 lux overhead light. Mice were shuttled to the center chamber, doors were closed, an empty wire cup was placed in the left chamber and a wire cup containing a conspecific mouse was placed in the right chamber. The doors were opened and mice were free to explore the three chambers for 10 min while being recorded by a video camera. An experienced researcher scored the duration sniffing the empty and social cup. The placement of empty cup and cup with conspecific mouse were counterbalanced across animals.

### Porsolt forced swim test

The forced swim test (FST) was performed as previously described (Cryan et al., 2002). For two consecutive days, mice were placed in a transparent 3 L plastic beaker containing 2.2 L of 25°C water for 6 min. Swimming activity was recorded automatically for 6 min and activity in the last 4 min was analyzed. Water was changed between subjects.

### Stress-induced corticosterone

Mice were placed in a clean novel cage for 30 min under 250 lux overhead light and blood was immediately drawn from the submandibular vein. Cortiocosterone levels were assessed by ELISA (Enzo Life Sciences).

### Chronic social defeat paradigm

The social defeat paradigm was performed as described (Golden et al., 2011) with modifications. Briefly, homecages were divided in half by a transparent perforated plastic divider (Animal Care Systems) and CD1 aggressor mice (Charles River Laboratories, Wilmington, MA) were housed on the right side of the divider and not moved for the 10 day duration of the experiment. For a social defeat session experimental mice were placed on the right side of a homecage with a CD1 for 10 min, then moved to the left empty side opposite the aggressor for 24 h. Once a day for 10 days mice experienced a social defeat session with a new CD1 resident aggressor. Control mice also lived in cages divided by a transparent perforated plastic divider and were moved to a new cage every day. On the last day of defeat or control conditions mice were placed into individual cages.

### Social interaction post-defeat

Twenty four hours following placement into individual cages social interaction was assessed as described with modifications (Golden et al., 2011) in a brightly lit room (250 lux). Mice were placed in a transparent Plexiglas open field (60 × 60 cm) opposite an empty upside-down wire cup and were allowed to explore for 180 s. Mice were removed from the open field for 30 s while an unfamiliar CD1 mouse was placed under a new wire cup. Experimental mice were then returned to the open field and allowed to explore for another 180 s. Sessions were recorded by video and an experienced researcher scored the duration mice sniffed the cup in both conditions and number sniff epochs. The social sociability score was calculated as the (amount of time spent sniffing the CD1 cup)/(amount of time spent sniffing the empty cup).

### Immunohistochemistry

Mice were anesthetized with a mixture of 150 mg/kg ketamine and 10 mg/kg xylazine and perfused transcardially with ice cold 0.9% saline followed by 4% paraformaldehyde (PFA). Brains were removed and stored in 4% PFA for 24 h, then switched to 30% sucrose solution for 48 h for cryoprotection. Brains were sectioned coronally on a cryostat at 35 μm, collected in serial sections of six and stored in PBS with 0.02% sodium azide.

For immunostaining, tissue was washed with PBS prior to incubation in 2 N HCl for 30 min at 37°C followed by 10 min in 0.1 M boric acid. Tissue was washed again in PBS, blocked with 10% normal donkey serum and incubated in primary antibody for 48 h at 4°C. The following primary antibodies were used: rat anti-BrdU (1:500, Accurate) for CldU, mouse anti-BrdU (1:1000, Becton Dickinson) for IdU, chicken anti-GFP (1:500, Santa Cruz) and Hoechst which served as a counterstain. All fluorescent secondary antibodies were obtained from Jackson ImmunoResearch and diluted 1:200 in PBS.

### Microscopy

Tissue was imaged at 20× on a fluorescent microscope (Olympus IX83) and CldU, IdU and GFP positive cells were identified and counted in the SGZ of the DG in a series of six coronal sections that traversed the septotemporal axis of the hippocampus.

### Statistics

All statistics were calculated by GraphPad Prism. All data are presented as the means ± SEM and significance was set at *p* < 0.05. Immunostaining, plasma corticosterone levels and social interaction post-defeat results were analyzed by *t*-test. Forced swim test was analyzed by two-way analysis of variance (ANOVA) with day and genotype as between subject variables. Sucrose preference test was analyzed by two-way ANOVA with day and genotype as between subject variables. Social affiliation was analyzed by two-way ANOVA with cup and genotype as between subject variables. ANOVAs that yielded statistically significant main effects were followed with Bonferroni *post hoc* tests.

## Results

### VGCV treatment transiently reduces cell proliferation when administered for 1 week in adolescence (P28-P35) or in adulthood (P56-P63)

**Figure 1:**
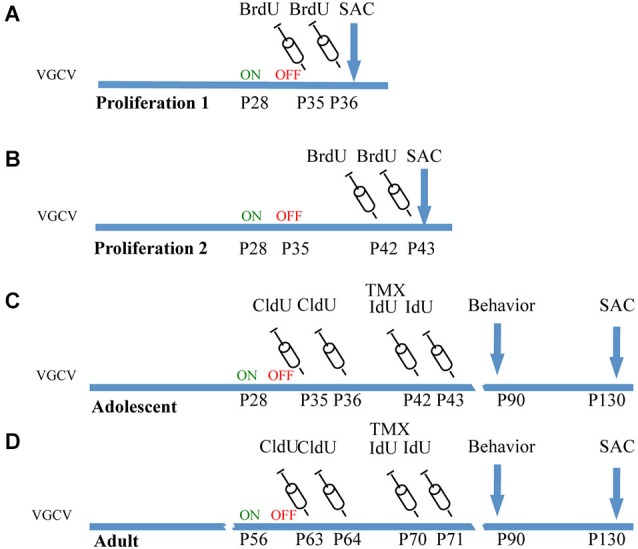
**Experimental cohorts of Nestin-CreERT2/R262R-Stop- EYFP/GFAP-Tk mice. (A)** Proliferation 1. To observe the effect of VGCV on cell proliferation, Tk^−/−^ and Tk^+/−^ mice were treated with VGCV from P28-P35, injected with cell proliferation marker BrdU on P35 and P36 and sacrificed on P36. **(B)** Proliferation 2. To observe cell proliferation 7 days following cessation of VGCV, Tk^−/−^ and Tk^+/−^ mice were treated with VGCV from P28-P35, injected with cell proliferation marker BrdU on P42 and P43 and sacrificed on P43. **(C)** Adolescent cohort of mice: Tk^−/−^ and Tk^+/−^ mice were treated with VGCV from P28-P35, cell proliferation marker CldU was injected on P35 and P36 to assess the effect of VGCV and proliferation marker IdU was injected on P42 and P43 to assess recovery following VGCV cessation. Tamoxifen (TMX) was administered on P42 to indelibly mark the nestin lineage in Cre^+^ mice. Behavioral testing was started on P90 and animals were sacrificed on P130. **(D)** Adult cohort of mice: Tk^−/−^ and Tk^+/−^ mice were treated with VGCV from P56-P63, cell proliferation marker CldU was injected on P63 and P64 to assess the effect of VGCV and proliferation marker IdU was injected on P70 and P71 to assess recovery following VGCV cessation. Tamoxifen (TMX) was administered on P70 to indelibly mark the nestin lineage in Cre^+^ mice. Behavioral testing was started on P90 and animals were sacrificed on P130.

**Figure 2:**
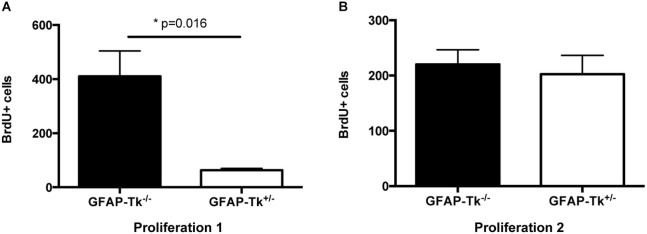
**Reduction and recovery of SGZ proliferation by VGCV administered P28-35. (A)** Proliferation 1. Fewer BrdU+ cells were present in GFAP-Tk^+/−^ mice immediately following VGCV treatment (BrdU injected P35 and P36, assessed P36; GFAP-Tk^−/−^ *n* = 2, GFAP-Tk^+/−^ *n* = 3). **(B)** Proliferation 2. Cell proliferation measured by BrdU is not affected 7 days following cessation of VGCV treatment in GFAP-Tk^+/−^ mice (BrdU injected P42 and P43, assessed P43; GFAP-Tk^−/−^ *n* = 3, GFAP-Tk^+/−^ *n* = 3). Bars represent mean ± SEM.

To observe the immediate effects of VGCV on cell division, a cohort of mice was administered VGCV from P28-P35, injected with BrdU on P35 and P36 and sacrificed on P36 (Figure 1A, Proliferation 1). A separate cohort of mice was administered VGCV from P28-P35 injected with BrdU on P42 and P43 and sacrificed on P43 (Figure 1B, Proliferation 2). After 7 days of VGCV treatment, the number of BrdU+ cells was reduced by 85% in GFAP-Tk^+/−^ mice (Figure 2A; *t*_(3)_ = 4.916, *p* = 0.016) but no differences in BrdU+ cells were detected 7 days following cessation of VGCV by *t*-test (Figure 2B). Therefore, cellular proliferation was dramatically reduced by 1 week VGCV treatment and returned to baseline within 1 week of discontinuing VGCV.

**Figure 3:**
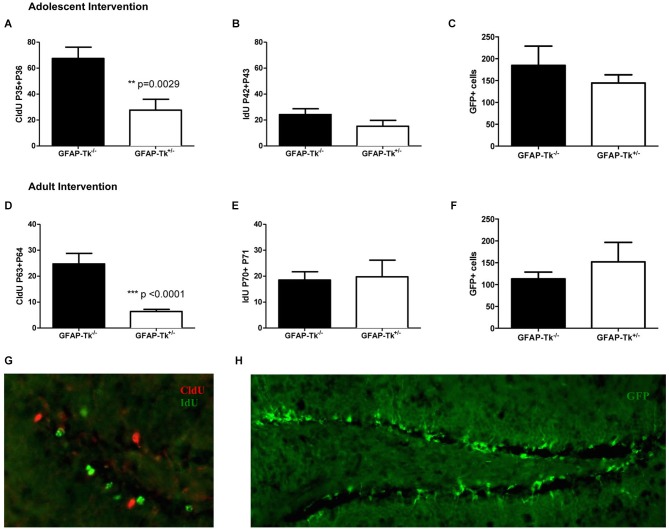
**Transient reduction of neurogenesis during adolescence and adulthood in Nestin-CreERT2/R26R-Stop-EYFP/GFAP-Tk mice by treatment with VGCV. (A)** Tk^+/−^ mice treated with VGCV during adolescence, P28-P35, show a reduction in proliferative marker CldU administered at the end of treatment and assessed in adulthood (Tk^−/−^ *n* = 17, Tk^+/−^ *n* = 14) **(B)** There is no change in proliferative marker IdU administered 7 days after termination of treatment and assessed at P130 (Tk^−/−^ *n* = 10, Tk^+/−^ *n* = 4) or **(C)** in the cumulative production of Nestin progeny labeled by GFP (Tk^−/−^ *n* = 7, Tk^+/−^ *n* = 8) indicating a reversible reduction in neurogenesis between P28 and P42. **(D)** Tk^+/−^ mice treated with VGCV during adulthood, P56-P63, show a reduction in proliferative marker CldU administered at the end of treatment and assessed at P130 (Tk^−/−^ *n* = 12, Tk^+/−^ *n* = 16). **(E)** There is no change in proliferative marker IdU administered 7 days after termination of treatment and assessed in adulthood (Tk^−/−^ *n* = 4, Tk^+/−^ *n* = 4) or **(F)** in the cumulative production of Nestin progeny labeled by GFP indicating a reversible reduction in neurogenesis between P56 and P70 (Tk^−/−^ *n* = 9, Tk^+/−^ *n* = 7). **(G)** Representative image of CldU labeled cells (green) and IdU labeled cells (red) in the SGZ. **(H)** Representative image of GFP labeling of nestin cell lineage. Bars represent mean ± SEM.

Mice used for behavioral experiments were treated with VGCV from P28-P35 in the adolescent group (Figure 1C, Adolescent) and P56-P63 in the adult group (Figure 1D, Adult). We used two thymidine analogs CldU and IdU, which can be identified by separate antibodies, to observe cells that divided when each of the compounds was administered (Figure 3G). We also used tamoxifen to label the cumulative neural stem cell lineage in Nestin-CreERT2 heterozygous R26R-Stop-EYFP homozygous mice (Figure 3H). In the adolescent cohort, the number of cells dividing at the end of VGCV treatment that survived until P130 was reduced by 60% in GFAP-Tk^+/−^ mice compared to GFAP-Tk^−/−^ littermate control mice (Figure 3A; *t*_(29)_ = 3.258, *p* = 0.0029). In adult mice the number of cells dividing at the end of VGCV treatment that survived until P130 was reduced by 75% in GFAP-Tk^+/−^ mice compared to GFAP-Tk^−/−^ littermate control mice (Figure 3D; *t*_(26)_ = 5.125, *p* < 0.0001). No difference in the number of IdU+ cells was detected between GFAP-Tk^+/−^ and GFAP-Tk^−/−^ mice in either adolescent or adult group by *t*-test (Figures 3B,F). In addition we observed that GFAP-Tk^−/−^ mice appeared to have a higher levels of neurogenesis at P35-P36 compared to P42-P43 and P63-P64 (Figures 3A,D). This reflects a well-documented age-related decline in neurogenesis (Ansorg et al., 2012) and may be further accentuated by the use of different labels (CldU and IdU) at the different time points. Neurogenesis remained constant in GFAP-Tk^−/−^ mice when assessed at P42-43, P63-64 and P70-P71 (Figures 3B,D,E). Finally no difference was observed in the number of GFP cells in GFAP-Tk^−/−^ and GFAP-Tk^+/−^ mice in adolescent and adult cohorts by *t*-test (Figures 3C,F) and as expected GFAP-Tk^−/−^ mice had a larger accumulation of cells in the nestin lineage when recombination was initiated at P42 compared to P70 (Figures 3C,F).

Put together in both adolescent and adult groups VGCV treatment in GFAP-Tk^+/−^ mice reduced markers of cell division after 7 days of treatment and markers of cell division were restored 7 days following VGCV cessation. Moreover we detected no differences in the numbers of cells resulting from lineage tracing of nestin stem cells in both adolescent (Figure 3C) and adult (Figure 3F) groups. We therefore transiently reduced neurogenesis for 1–2 weeks specifically during adolescence and adulthood.

### Reduction of neurogenesis in adolescence and adulthood does not influence depression-related behavior, social affiliation or corticosterone response to an acute stressor

**Figure 4:**
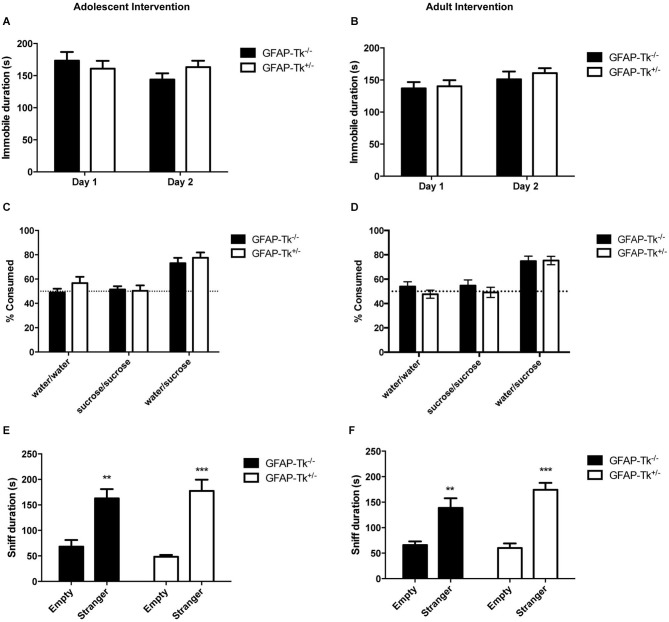
**Depression-related behavior in adult mice was not affected by transient reduction of neurogenesis during adolescence or in adulthood**. No differences were detected between GFAP-Tk^−/−^ and GFAP-Tk^+/−^ mice in immobility in the forced swim test in the **(A)** adolescent or **(B)** adult cohorts. GFAP-Tk^−/−^ and GFAP-Tk^+/−^ mice in **(C)** adolescent and **(D)** adult groups demonstrated similar preference for a sucrose solution when presented with a choice. No preference for either bottle was observed when no choice between water and sucrose was given. GFAP-Tk^−/−^ and GFAP-Tk^+/−^ mice in both the **(E)** adolescent and **(F)** adult groups displayed a preference to explore a stranger compared to an empty cup, **p < 0.01, ***p < 0.001 stranger compared to empty cup. No difference between Tk^−/−^ and Tk^+/−^ was detected. Bars represent mean ± SEM. For FST and sucrose adolescent cohort GFAP-Tk^−/−^ *n* = 25, GFAP-Tk^+/−^ *n* = 27, adult cohort GFAP-Tk^−/−^ *n* = 21, GFAP-Tk^+/−^ *n* = 29. For social affiliation adolescent cohort GFAP-Tk^−/−^ *n* = 8, GFAP-Tk^+/−^ *n* = 12, for adult cohort GFAP-Tk^−/−^ *n* = 6, GFAP-Tk^+/−^ *n* = 10.

**Figure 5:**
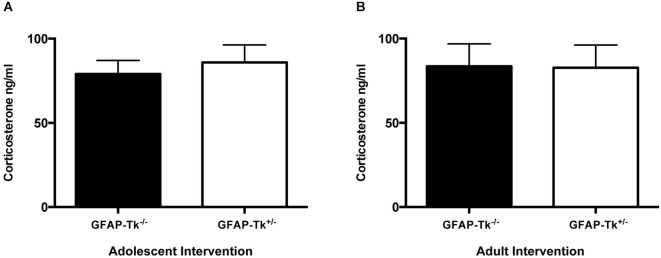
**Following a mild stressor, novel cage exposure, differences in plasma corticosterone were not detected between GFAP-TK^−/−^ and GFAP-Tk^+/−^ mice in (A) adolescent or (B) adult groups.** Bars represent mean ± SEM for adolescent cohort GFAP-Tk^−/−^ *n* = 15, GFAP-Tk^+/−^ *n* = 18, Adult cohort GFAP-Tk^−/−^ *n* = 13, GFAP-Tk^+/−^ *n* = 17.

To test if reductions in neurogenesis during adolescence and adulthood influence depression-related behavior, mice were tested in three behavioral measures related to depression and antidepressant response. The Porsolt forced swim test is sensitive to antidepressant treatment and is thought to measure passive stress coping or behavioral despair in rodents (Porsolt et al., 1977). We previously showed that baseline FST behavior is altered in mice genetically altered to model depression (Richardson-Jones et al., 2010). However, a reduction in neurogenesis in adolescence or adulthood did not influence behavior in this task in adult mice. No differences between groups were detected in immobile duration on day 1 or day 2 of the forced swim test in adolescent (Figure 4A) or adult (Figure 4B) groups by two-way ANOVA. The sucrose preference test is also sensitive to antidepressant treatment and assesses the hedonic drive in adult mice. Reduction in neurogenesis during adolescence or in adulthood did not influence hedonic state in adult mice. When mice with reductions in adolescent and adult neurogenesis were presented with either two identical water bottles (water/water) or two identical bottles containing 4% sucrose (sucrose/sucrose), they consumed fluid equally from the two bottles (Figures 4C,D). However when mice were presented with one bottle of water and one bottle containing 4% sucrose (water/sucrose), both GFAP-Tk^−/−^ and GFAP-Tk^+/−^ mice from adolescent and adult cohorts preferred the bottle containing sucrose. No differences in preference to consume sucrose were detected between GFAP-Tk^−/−^ and GFAP-Tk^+/−^ mice in either the adolescent or adult cohort by two-way ANOVA. Mice from all groups consumed approximately 75–80% sucrose solution. To assess social affiliation mice had an opportunity to explore an empty wire cup or a cup containing a stranger conspecific mouse. We did not detect an effect of reducing neurogenesis in adolescent or adult groups as all mice showed a strong preference to explore a conspecifc mouse compared to an empty cup (Figures 4E,F; main effect of cup *F*_(1,36)_ = 46.38, *p* < 0.0001, main effect of cup *F*_(1,28)_ = 50.71, *p* < 0.0001). Finally exposure to a novel cage activates the HPA axis above baseline resulting in an increase of corticosterone into the bloodstream (Schloesser et al., 2009). Plasma levels of corticosterone were measured in mice following exposure to a novel cage for 30 min. We did not detect differences in corticosterone levels in adult mice with neurogenesis reduced during adolescence or adulthood by *t*-test (Figures 5A,B).

### Reduction of neurogenesis in adolescence but not adulthood promotes resilience to social defeat

**Figure 6:**
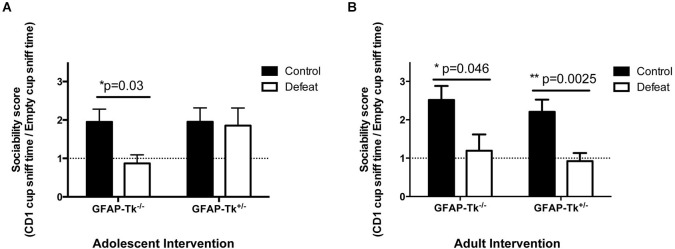
**Susceptibility to social defeat. (A)** In the adolescent group GFAP-Tk^−/−^ mice showed a reduced sociability score following social defeat compared to control (naïve) mice indicating susceptibility. The sociability scores of GFAP-Tk^+/−^ mice following social defeat do not differ from GFAP-Tk^+/−^ or GFAP-Tk^−/−^ naïve mice indicating resilence. **(B)** In the adult group both GFAP-Tk^−/−^ and GFAP-Tk^+/−^ mice in the defeat condition show reduced sociability scores following social defeat compared to naïve mice indicating susceptibility. Bars represent mean ± SEM, for adolescent cohort GFAP-Tk^−/−^ control *n* = 8, GFAP-Tk^−/−^ defeat *n* = 8, GFAP-Tk^+/−^ control *n* = 8, GFAP-Tk^+/−^ defeat *n* = 8. Adult cohort GFAP-Tk^−/−^ control *n* = 8, GFAP-Tk^−/−^ defeat *n* = 9, GFAP-Tk^+/−^ control *n* = 10, GFAP-Tk^+/−^ defeat *n* = 12.

To assess responses to chronic stress mice underwent a chronic social defeat paradigm. Control groups naïve to social defeat were also used in each case. During the social defeat paradigm we observed similar levels of aggression in the CD1 mice across the 10 min defeat sessions so each experimental mouse received comparable treatment. In the adolescent cohort, GFAP-Tk^−/−^ and GFAP-Tk^+/−^ mice in the naïve group preferred to explore a wire cup containing a CD1 mouse compared to an empty cup thus had sociability scores >1 (Figure 6A). GFAP-Tk^−/−^ mice exposed to social defeat did not show a preference to explore a wire cup containing a CD1 mouse thus exhibiting significantly reduced sociability scores compared to the naïve group (Figure 6A; *t*_(13)_ = 2.412, *p* = 0.031). However, similar to the naïve group, GFAP-Tk^+/−^ mice exposed to social defeat preferred to explore the CD1 mouse (Figure 6A), demonstrating a resilient phenotype. In the adult group, both GFAP-Tk^−/−^ and GFAP-Tk^+/−^ mice in the defeat condition showed reduced sociability scores compared to the control condition (Figure 6B; *t*_(13)_ = 2.206, *p* = 0.046, *t*_(20)_ = 3.460, *p* = 0.0025) indicating a susceptible phenotype as expected.

## Discussion

We successfully applied a 7-day treatment of VGCV in GFAP-Tk^+/−^ mice to transiently reduce neurogenesis during adolescence or adulthood. We observed that VGCV treatment in GFAP-Tk^+/−^ mice reduced cell proliferation in the SGZ when assessed immediately following treatment. Consequently, fewer of these cells were present in adult animals. The reduction in neurogenesis was reversed 1 week following cessation of VGCV as levels of both cell proliferation and the survival of proliferating cells were restored and the cumulative nestin lineage was unaffected. By developing a method to transiently reduce neurogenesis at discrete developmental stages we demonstrated that cells born during adolescence contribute differently to behavior than cells born in adulthood. Applying our method to other significant developmental time points will be informative.

We found that a reduction of proliferating cells during adolescence leads to resilience following chronic social defeat in adulthood. This phenomenon appears to be independent of emotional systems mediating three discrete dimensions of depression: despair, hedonic drive and sociability. Our results are informative in regards to the neurocircuitry of depression and stress resilience. The resilient GFAP-Tk^+/−^ mice treated with VGCV during adolescence do not show behavioral changes at baseline related to despair (FST), reward circuitry (sucrose preference) or sociability (social affiliation) while displaying resilience following social defeat. This outcome suggests that stress resilience is dissociable from baseline depressive-like behavior and reward circuitry. Similarly, a recent study demonstrated that anxiety-like behavior and stress resilience following social defeat might be supported by disparate circuitry (Iniguez et al., 2014). Thus mechanisms of chronic stress susceptibility or resilience may be independent of those regulating baseline anxiety, despair and reward. We also observed that the baseline response to an acute mild stressor is independent of the resilient phenotype, as corticosterone responses in adults were unaffected by our intervention in the adolescent cohort. These results suggest that the contribution of adolescent-born neurons to chronic stress susceptibility is independent of the involvement of neurogenesis in responses to acute stress or to depressive-like behaviors. Analysis of anxiety and depression related behaviors and corticosterone following chronic social defeat after similar targeted interventions would further inform this possibility.

There are a few caveats that should be considered in interpreting our study. It is important to note that neurogenesis is suppressed in both the SGZ and the subventricular zone (SVZ) in GFAP-Tk+ mice treated with VGCV. Neural stem cells in the SVZ differentiate into interneurons that integrate into the main olfactory bulb (Zhao et al., 2008). Unlike the hippocampus (Tsankova et al., 2006), the olfactory bulb has not been implicated in responses to social defeat. However, it is possible that adolescent-born olfactory bulb neurons partially contribute to our phenotype. Future studies utilizing more restricted approaches such as targeted x-irradiation of the hippocampus or the rostral migratory stream will be needed to study this directly. Next, the age of the neurons ablated in adolescent and adult cohorts differ by 4 weeks at the time of behavioral testing. Specifically, during social defeat the adolescent cohort was missing 13–15 week old cells, while the adult cohort was missing cells 9–11 week old cells. Thus it is possible that 13–15 week old cells are involved in producing susceptibility following social defeat while 9–11 week old cells are not. However, most studies of granule cell maturation support the notion of near full maturity by 8 weeks after cell birth. For example, granule cells older than 7 weeks that differ in age by 4 weeks show functional homogeneity (Laplagne et al., 2006). While we are not aware of reports suggesting functional segregation between 9–11 week old and older cells it remains a possibility.

A few mechanistic hypotheses emerge from our result that a reduction in neurogenesis during adolescence leads to resilience. The resilient phenotype could arise: (1) directly from a reduction in the total number of cells that comprise DG; (2) from a reduction of a specific specialized population of cells that develop during adolescence; or (3) by circuit-level adaptation. Firstly, since neurogenesis is significantly higher in adolescent mice than adult mice, our intervention reduced more cells in adolescents. Therefore, resilience in the adolescent cohort could have resulted from a larger loss of total cells in the DG. Alternatively, we may have eliminated a specialized population of DG cells that are functionally distinct from adult born cells and thereby preferentially involved in chronic defeat stress. One prior report suggested that permanent reduction of neurogenesis by whole brain x-irradiation, in mice between P35-P56, leads to a resilient phenotype 4 weeks later (Lagace et al., 2010). The findings described here indicate that the effects seen by Lagace and colleagues are largely due to the loss of adolescent-born, but not adult-born cells. Our results therefore argue for the exciting possibility that the seemingly homogeneous population of DG granule cells is functionally diverse and that diversity is specified by the ages of the cells. Finally, the adolescent born cells we have eliminated with our intervention could be preferentially maintaining brain circuits responsible for sensing, encoding or expressing defeat susceptibility. The DG receives input from many brain structures (Leranth and Hajszan, 2007) and may regulate CA3 output to an equally diverse range of brain regions (Tannenholz et al., 2014). However, the importance of DG granule cells in maintaining mature circuits remains unexplored.

Consequences of chronic social defeat are encoded in the ventral tegmental area, nucleus accumbens (Cao et al., 2010; Chaudhury et al., 2013), prefrontal cortex (Covington et al., 2010) and the hippocampus (Tsankova et al., 2006). Extensive connectivity between these brain regions highlights the importance of a systems level understanding of how the four structures interface in contributing to encoding susceptibility to chronic stress. Remarkably all four of these brain regions undergo extensive circuit maturation during adolescence (Casey et al., 2008; Pattwell et al., 2011). A decrease in neurogenesis by our intervention could therefore impact hippocampal connections and possibly downstream brain regions developing during this sensitive period. Since stress significantly reduces SGZ neurogenesis, stress during adolescence may act by reducing neurogenesis to alter its connectivity and prepare the animal for buffering chronic stress in adulthood.

## Conflict of interest statement

The Guest Associate Editor Mazen A. Kheirbek declares that, despite being affiliated to the same institution as the authors, the review process was handled objectively and no conflict of interest exists. The authors declare that the research was conducted in the absence of any commercial or financial relationships that could be construed as a potential conflict of interest.
